# Evaluation of integrated modular teaching in Chinese ophthalmology trainee courses

**DOI:** 10.1186/s12909-020-02073-w

**Published:** 2020-05-19

**Authors:** Wei Xin, Yuxian Zou, Yong Ao, Yu Cai, Zheqian Huang, Miaoling Li, Chaochao Xu, Yu Jia, Ying Yang, Yangfan Yang, Haotian Lin

**Affiliations:** 1 State Key Laboratory of Ophthalmology, Zhongshan Ophthalmic Centre, Sun Yat-sen University, Guagzhou, 510060 Guangzhou, People’s Republic of China; 2 Teaching Reform Research Group, 7#, Jinsui Road, Guangzhou, 510000 China

**Keywords:** Ophthalmology, Teaching, Effectiveness, Problem-based, Trainee course

## Abstract

**Background:**

Before attending ophthalmology trainee courses in Zhongshan Ophthalmic Centre, the medical students from Sun Yat-sen University had finished two years of premedical education after the six-year medical courses including basic medical courses, clinical medical courses, clerkship, and research training in medical college. Integrated modular teaching using different problem-based teaching methods in ophthalmology was designed by the teaching steering committee of Zhongshan Ophthalmic Centre. This study aimed to evaluate the effectiveness and satisfaction scales of the integrated modular teaching among the trainee students.

**Methods:**

A total of 100 medical students attending ophthalmology trainee courses in Zhongshan Ophthalmic Centre were enrolled and randomly allocated into 4 groups according to the teaching arrangement. The trainee courses consisted of several sessions delivered in multiple methods, such as “flipped classroom” session and team-based learning session. The pre- and post-class tests were delivered to evaluate the effectiveness of the integrated modular teaching. The satisfaction survey questionnaire was collected from all participants to investigate the degree of satisfaction.

**Results:**

Compared with the first-day-test score, the total last-day-test score was significantly improved by a paired *t-*test (*t* = 3.288, *P* = 0.001). Nineteen students obtained a significant improvement in ranking increased by more than 10 in the last-day-test, whereas they failed to obtain a higher average score for daily performance than other students (*t* = 0.469, *P* = 0.654). According to the participant satisfaction questionnaires, these innovative teaching methods were considered as effective and satisfactory.

**Conclusions:**

Integrated modular teaching in ophthalmology trainee courses is effective and appreciated by the medical college students.

## Background

Ophthalmology is an independent and essential subject in medical teaching in which clinical-skill training and patient interaction play indispensable roles [1]. However, many medical students lack of sufficient practice in ophthalmology due to insufficient teaching time [2, 3]. In addition, the teaching style that most medical colleges employ in teaching ophthalmology may lack efficiency and student interest. With the advancement in science and technology and multimedia innovations over the last several decades, new teaching methodologies have been introduced into medical teaching to improve the learning efficiency, shorten classroom learning time and develop students’ ability for autonomous learning. Because ophthalmology is a discipline that emphasizes practice, multimedia teaching method should be adopted to demonstrate a variety of ocular pictures and videos and to emphasize the items of information repeatedly.

“Flipped classroom” (an inverted model of teaching that uses videos, podcasts or slides to deliver lecture materials outside the classroom, therefore, the classroom time is mainly spent on discussion or problem-solving [4]), team-based learning (TBL) [5], simulation scenarios [6], figure demonstrations [7] and video demonstrations [8] have been shown as effective teaching methodologies to promote the transformation of teaching from a teacher-centred approach to one that focuses on medical students. The use of TBL has been applied in ophthalmology teaching and has proven to be an effective means of active learning [9]. However, most teaching assessments have been implemented based on one or two teaching methodologies [10–13]. Most subjects, including ophthalmology, seldom use a single model for teaching. Instead, a multi-mode teaching model is frequently employed in the actual teaching process. Because every student has a preferred learning mode, it is necessary to integrate a variety of teaching modes to satisfy the students’ needs and improve their learning efficiency. Recently, integrated modular teaching using different teaching methodologies has yielded improved results in dermatology [7].

In the present study, 100 medical students from Sun Yat-sen University took part in an ophthalmology curriculum, which was designed to incorporate multiple innovative teaching methodologies. This study was designed to assess the effectiveness and the students’ degree of satisfaction among different innovative teaching methods, aiming to evaluate the validity and feasibility of integrated modular teaching in ophthalmology by using pre-, post-class tests and daily performance.

## Methods

### Participants

In this prospective study, 100 medical students who were entering their ninth semester in August 2015 and taking classes in Sun Yat-sen University were enrolled. All students were randomly allocated into 4 groups in order to guarantee the teaching quality. The students participated in a 1-week of integrated modular ophthalmology learning course in Zhongshan Ophthalmic Centre. All the procedures in this study were videotaped, with the approval of the institutional review board of Zhongshan Ophthalmic Centre of Sun Yat-sen University (IRB-ZOC-SYSU) (Ethic ID: 2016MEKY062). Written informed consents have been obtained from all students.

### Integrated modular teaching

Each module was initiated by a clinical teacher from Zhongshan Ophthalmic Centre with a suitable introduction, followed by a discussion of individual topics. Pre- and post-class tests were conducted at the beginning of the first module and the end of the last module, respectively, to assess the effectiveness of the integrated modular teaching system. Test papers were designed based on the teachers’ discussions and approved by the examination administration of Sun Yat-sen University.

The topics of these modules included the anatomy of the eye, ophthalmologic examination, the lids and lacrimal apparatus, conjunctivitis and uveitis, keratitis, glaucoma, cataract and refractive error, the retina and ocular disorders associated with systemic disease, ocular trauma and blindness (Table 1).

**Table 1:** Modular teaching on topics of ophthalmology and teaching methodologies

Teaching methodology	Module	Description
Lecture	Anatomy of the eye	Systematic introduction to sensitize and familiarize the student with certain concepts and clinic features through PPT
	Ophthalmologic examination	
	Lids and lacrimal apparatus	
	Cataract and refractive error	
	Retina and ocular disorders associated with systemic disease	
Video demonstration	Anatomy of the eye	Video demonstration of all the steps of a procedure
	Retina and ocular disorders associated with systemic disease	
Practice	Anatomy of the eye	Basic ophthalmic examination practice and dissecting a pig eyeball
	Ophthalmologic examination,Cataract and refractive error	
Photo demonstration	Lids and lacrimal apparatus	A series of images of various manifestations of the disease were demonstrated by projection. The students had to describe what they saw and attempt to explain the correlation with the clinical condition
	Retina and ocular disorders associated with systemic disease	
Case-based learning (CBL)	Conjunctivitis and uveitis, Keratitis	Typical cases from outpatient clinic or hospital ward were demonstrated. The students had to inquire about medical history, collect clinical examination information and attempt the diagnosis. A summary of key learning points was given by the clinical teacher
Simulation scenario teaching	Glaucoma	Clinic teachers and students play patients with ocular disease and doctors in a scenario, o, respectively. Students acted as doctors and asked questions. A summary of key learning points was given by the clinical teacher.
Flipped classroom	Ocular trauma	Some problems were raised from the learning material by the teacher. The students had to self-study all content, answer all questions and provide new questions for the discussion in class.
Team-based learning (TBL)	Blindness	Students of each group had to complete a presentation associated with the given topic.

A comprehensive evaluation was conducted among all the enrolled students based on the comparison between the first-day and last-day test scores before and after 1-week of integrated modular teaching with innovative teaching methods. The details of the questionnaire utilized in the comprehensive evaluation are illustrated in Supplementary materials. The questionnaire consisted of two parts. The first part was a case with chief complains of single eye redness and blurred vision. Three questions about important signs, diagnosis and recommended examinations of this case were listed to be answered. The second part contained a list of important ocular symptoms and signs, such as photophobia, tearing, itch sensation, foreign-body sensation and swelling pain, etc. Five cases were presented in the 2nd part and the students were asked to select one or more related symptoms or signs for each case.

### “Flipped classroom” session

The “flipped classroom” protocol was used for the ocular trauma teaching among the fifth-year medical college students [14]. Before the “flipped classroom”, relevant knowledge and questions based on one case of single ocular trauma were delivered by email. The students were asked to overview the knowledge of ocular trauma in groups using textbooks, published articles, e-publications or any other reference besides the email.

At 3 days before the “flipped classroom” session, the students were directed to review the subject of ocular trauma. One test consisting of three cases was completed by students in 20 min at 1 day before the “flipped classroom” session. These three cases focused on penetrating injuries, contusions of the eyeball and chemical burns. Two or three questions were appended to each case scenario. Before the end of the course, a post-class test was administered immediately to obtain feedback. Simultaneously, a questionnaire was finished by each student to investigate the satisfaction scale.

### Team-based learning module

The TBL module [15] was designed according to the guidance [16], which included Individual Readiness Assurance Test (IRAT), preparatory assignments, Group Readiness Assurance Test (GRAT), Group Application Problem (GAP) and Final Examination Scores (FESs). In each group, students were given with a random topic in the morning of the first-day trainee course. The topics were closely linked to the content in this curriculum. The students had to prepare any form of presentation within 5 days according to the assigned topic. Other relevant medical knowledge in addition to ophthalmology was permitted to be discussed in the presentation or display. After students in each group presented their team-work presentations in class, the teachers graded the students’ presentations into 5 criteria.

### Statistical analysis

The pre- and post-class-test scores, and the first- and last-day-test scores were statistically compared by using paired *t*-test at the significance level of 0.05. One-way analysis of variance (ANOVA) was used to compare the average scores among different groups or teaching methods. All data analyses were performed using SPSS 20.0 statistical software (SPSS Inc., Chicago, IL, USA).

## Results

### Baseline data

A total of 100 students, 50 male and 50 female, aged from 22 to 28 years old, were recruited. The following information was collected from the student record department of the university: 1) The admission scores for the medical college were collected and no significant difference was observed in the admission scores among four groups (*F* = 0.520, *P* = 0.669), 2) The final grade for all curricula, which are provided as a standard numerical grade (SNG) on a scale from 0 to 100, including the following subjects: Obstetrics and Gynecology, Pediatrics, Psychiatry, Neurology, Emergency Medicine, Disaster Medicine, Otolaryngology Head and Neck Surgery, Anesthetics, Dermatovenerology, Internal Medicine, Surgery, and Ophthalmology, and there was no significant difference in the final grade for all curricula among four groups, 3) The final grade of ophthalmology theory did not significantly differ among four groups.

### Completion rate

All the 100 clinical medical students who took part in the ophthalmology trainee courses completed pre- and post-class tests in the integrated modular teaching provided by Zhongshan Ophthalmic Centre. All data were collected and analysed. Eight clinical teachers jointly completed the innovative teaching work. No complaints from the students were reported throughout the trainee process.

### Effectiveness of TBL and “flipped classroom” sessions

**Fig. 1:**
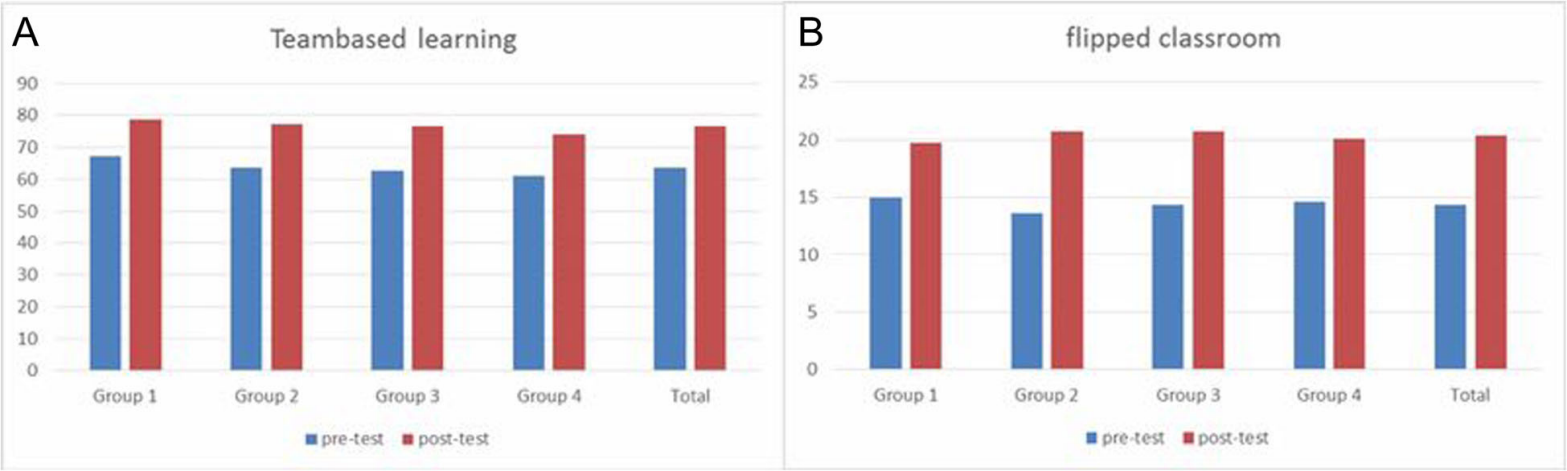
Average scores of the pre-class test and post-class test for team based learning (**a**) and flipped classroom (**b**) among the four groups and in total

The average scores of pre- and post-class tests in both TBL and “flipped classroom” sessions were calculated and analyzed. The pre- and post-class test scores in each group were statistically compared by using a paired *t-*test. The total students’ scores in each group were improved in the post-class test during both TBL and “flipped classroom” sessions (TBL: *t* = 12.208, *P* = 0.000; “flipped classroom”: *t* = 37.822; *P* = 0.000) (Fig. 1). The students’ questionnaires were collected and their acceptance of these two new teaching methods was evaluated by calculating the number of students who responded with “agree” (Table 2). Among them, 70% of the students believed that these two teaching methods were beneficial for learning. The details and survey questions of student perceptions regarding various teaching methods are presented in Table 2.

**Table 2:** Survey of Student Perceptions Regarding Two Teaching Methods

	Number of Strongly Agree/Agree Responses (%)	
Survey Question	Flipped classroom	Team-based learning
It helped me to reach a higher level of knowledge	93.30	89.41
It was an effective, motivating learning process	89.90	83.53
It was well organized	100.00	94.12
It challenged me to give my best	95.00	81.18
It had a positive impact on my learning attitudes	93.20	78.82
I was satisfied with this teaching method	89.80	75.29
It should be offered more frequently	81.40	75.29

### Comprehensive evaluation of the integrated modular teaching

**Fig. 2:**
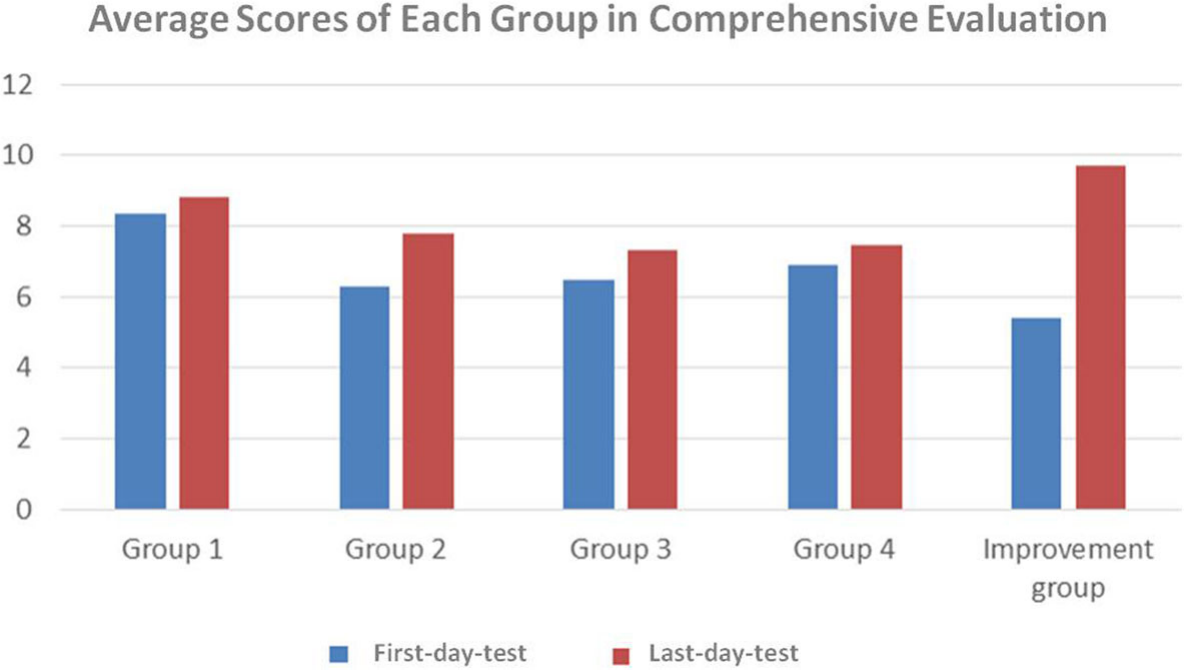
Average scores of each group in comprehensive evaluation. We picked out 19 students who had an increase in academic performance by more than 10 grades in the post-test, which were called improvement group

A comprehensive evaluation was conducted among all enrolled students by the first-day-test and last-day-test, which were organized before and after the integrated modular teaching with innovative teaching methods for 1 week. The average scores of each group, including the first-day-test and last-day-test, are presented in Fig. 2. The first-day-test scores did not significantly differ among four groups by ANOVA test. The paired *t-*test was used to compare the average score of first-day-test before the trainee courses and the total last-day-test score after the courses. The total last-day-test score was significantly improved (*t* = 3.288, *P* = 0.001). By comparing the first-day-test and last-day-test scores, 19 students who had outstanding performance with ranking increased by more than 10 in the post-class test were screened. There students were then assigned into the improvement group in Fig. 2, and the daily performance of these outstanding students was assessed to discuss whether they paid more attention in the classroom. However, they did not have higher average scores in daily performance than other students (*t* = 0.469, *P* = 0.654). Most students were satisfied with the innovative teaching methods based on their answers to the questionnaire. A small number of students did not agree with the effectiveness of these new teaching methods, even if their test results were improved.

## Discussion

With the introduction and development of multimedia, computers and the internet in this information era, modern technologies can increasingly be used to replace the previous teaching methods and to usher in an era of rich teaching resources. The applications of modern technology and abundant public learning resources have promoted a reform from previous teacher-centred to student-centred teaching modes. Student-centred teaching can cultivate higher-order thinking, problem solving and critical analysis, and provide feedback on the learning process [17]. Learning is a complex process, and previous studies have confirmed that increasing active learning can produce a better learning result [18, 19]. Recently, a variety of student-centred teaching methods have been conducted in medical teaching [10, 20].

The trainee courses of ophthalmology are established to improve students’ knowledge in clinical practice, to guide them to integrate knowledge in theory with practice, and eventually to attract more outstanding students to the ophthalmology profession. All the teaching modules we are conducting are based on problem-solving.

In this study, the effectiveness of each major innovative teaching method was evaluated by pre- and post-class tests for 100 medical students. The post-class scores of all students were improved. A majority of students were satisfied with the innovative teaching methods based on their answers to the questionnaire. Because each student has preferred learning habits and thinking modes, it is understandable that a small number of students do not agree with the effectiveness of these new teaching methods, even if their test results are improved. Overall, integrated teaching modes are appropriate for the learning habits of most students.

A comprehensive evaluation of integrated teaching modes was conducted by comparing the pre- and post-class test scores. Most students presented with significant improvement in the post-class test scores, suggesting that this multi-mode teaching method is highly effective in ophthalmology trainee courses. In previous teaching styles, the teacher-centred teaching method forces students to passively accept knowledge. The grades of students depend on how they listen in the classroom and review the materials after class. Diligent students can often obtain better grades. Therefore, different learning attitudes among students may make an enormous difference in the degree of knowledge they master. However, these innovative teaching methods are implemented based on student-centred teaching, which stimulates autonomous and active learning of the students. These curricula were designed to comprise different modules using a variety of teaching methods, which can enhance learning interests, reduce boredom and increase the acceptance by students.

The multi-mode teaching methods in this study included a “flipped classroom” session, consisted of simulation scenario teaching, case-based learning, and other methods that improved the autonomous learning capability of the students, which plays a pivotal role in the life-long career growth of medical students [21]. Therefore, the education of medical students is not only involved with teaching professional knowledge but also how to take the initiative to study and how to use the educational platforms provided by modern science and technology. In addition, the teamwork capability is of significance for medical students because hospitals are workplaces based on teamwork [22]. The TBL method can teach students how to learn from each other and jointly progress in a team.

Compared with other medical subjects, ophthalmology is more closely associated with advanced science and technologies, such as the application of lasers, new materials, and optical instruments, which require ophthalmologists to maintain effective learning and to develop more autonomous learning than other specialists. Therefore, this teaching method might provide particular assistance for medical students who are determined to become ophthalmologists.

In our study, the students’ daily performance was analysed. All students had worse daily performances in TBL session than the “flipped classroom” and case-based learning sessions, which was consistent with the questionnaire results. TBL had a lower degree of recognition. A total of 19 students obtained an increase in academic performance by more than 10 points in the post-class test, whereas their daily performance in class or attendance were not better compared with other students, probably prompting that all students have excellent class performance when autonomous learning awareness is triggered. This situation differs from previous learning modes because greater efforts have been made throughout the whole learning process, and more significant improvement has been obtained. Nevertheless, whether the learning effect will decrease over time remains elusive. Hence, these students should be followed up to examine their learning ability and the durability of this method in subsequent investigations.

## Conclusion

In the present study, the integrated modular teaching is an effective teaching method and appreciated by medical college students in ophthalmology trainee courses. It deserves to be introduced to the trainee or teaching process in other medical specialized subjects.

## Data Availability

The datasets used and/or analysed during the current study are available from the corresponding author on reasonable request.

## Abbreviations

TBL: Team-based learning
CBL: Case-based learning
ANOVA: One-way analysis of variance
